# Optimal Sensor Placement for Measuring Physical Activity with a 3D Accelerometer

**DOI:** 10.3390/s140203188

**Published:** 2014-02-18

**Authors:** Simone T. Boerema, Lex van Velsen, Leendert Schaake, Thijs M. Tönis, Hermie J. Hermens

**Affiliations:** 1 Telemedicine Group, Roessingh Research and Development, P.O. Box 310, 7500 AH, Enschede, The Netherlands; E-Mails: l.vanvelsen@rrd.nl (L.V.); l.schaake@rrd.nl (L.S.); t.tonis@rrd.nl (T.M.T.); h.hermens@rrd.nl (H.J.H.); 2 Telemedicine Group, Faculty of Electrical Engineering, Mathematics and Computer Science, University of Twente, P.O. Box 217, 7500 AE, Enschede, The Netherlands

**Keywords:** activity monitoring, waist-mounted, 3D accelerometer, physical activity, ambulatory assessment, calibration, reliability, treadmill, healthcare engineering

## Abstract

Accelerometer-based activity monitors are popular for monitoring physical activity. In this study, we investigated optimal sensor placement for increasing the quality of studies that utilize accelerometer data to assess physical activity. We performed a two-staged study, focused on sensor location and type of mounting. Ten subjects walked at various walking speeds on a treadmill, performed a deskwork protocol, and walked on level ground, while simultaneously wearing five ProMove2 sensors with a snug fit on an elastic waist belt. We found that sensor location, type of activity, and their interaction-effect affected sensor output. The most lateral positions on the waist belt were the least sensitive for interference. The effect of mounting was explored, by making two subjects repeat the experimental protocol with sensors more loosely fitted to the elastic belt. The loose fit resulted in lower sensor output, except for the deskwork protocol, where output was higher. In order to increase the reliability and to reduce the variability of sensor output, researchers should place activity sensors on the most lateral position of a participant's waist belt. If the sensor hampers free movement, it may be positioned slightly more forward on the belt. Finally, sensors should be fitted tightly to the body.

## 1. Introduction

Accelerometer-based activity monitors are currently the most widely used sensors for monitoring physical activity in clinical and free-living settings [1–3]. They can be used for monitoring physical activity to acquire more fundamental knowledge of patterns of physical activity or to generate input for health interventions. For the latter application, activity sensor data is used to determine performance and, subsequently, to provide real-time personalized feedback (e.g., for patients with Chronic Fatigue Syndrome [4] or COPD [5] to increase self-awareness and support behavior change. Studies on the implementation of 3D (tri-axial) accelerometer-based activity monitoring in healthcare have focused predominantly on overall behavior change or on clinical parameters on a group level [4,6]. One could assume that averaging sensor data over large populations or over time reduces the effects of usage and other non-controllable factors during free living. However, the shift towards individual programs on physical activity patterns makes this assumption no longer valid [7,8]. To increase the reliability and the validity of monitoring studies the influence of sensor placement and attachment has to be determined. However, the influence of the placement of the activity sensor itself on sensor output has not been studied in-depth before [8,9].

Most studies in which activity counts or Energy Expenditure (EE) are used as a primary outcome measure, place a single sensor at the lower back (sacrum) or at the waist—close to the center of mass of the human body. These sensors can be directly attached to the skin or indirectly attached by using belts, clips or other accessories [9–11]. As the movement of clothes can cause interference in the accelerometer output, Bouten *et al.* [10] validated the tri-axial Tracmor monitor for predicting EE with the sensor attached directly to the skin. This placement hampered usability and increased subject burden, especially over prolonged periods of time, and in later validation-studies we can see that the sensor was no longer attached directly to the skin, but worn using an elastic belt [11]. Researchers have to choose between minimizing the relative motion between the sensors and the human body on the one hand (by providing a snug fit of the sensor against the body), and maintaining a high level of usability and comfort on the other [9]. But in order to be able to make this decision, they should know the effect of sensor placement on sensor output.

With this study, we aim to improve the quality of accelerometer sensor output for laboratory-based and free-living studies. We have conducted a two-staged study that resulted in concrete guidelines for wearing an activity sensor that increases reliability and reduces the variability of output data without compromising usability and comfort.

## 2. Background

3D accelerometer-based activity monitors are small, lightweight, portable, non-invasive, and non-intrusive devices that record motion in three planes and provide an indication of the intensity level of physical activity [12]. In the last few years, research with activity monitors is becoming more uniform with the growing availability of assessment guidelines and best practices.

### 2.1. Current Guidelines for Research with Wearable Monitors

Current guidelines for assessing physical activity using wearable monitors focus on both sensor calibration and practical use [3,13–16]. These guidelines are based on the latest research evidence and on the consensus of researchers in the field of objective monitoring of physical activity. They recommend that researchers provide the rationale for the selection of a particular monitor, like its reliability and validity for the target population. This rationale should also include a description of how the monitor was positioned on the participant during the calibration and validation studies [13].

Most guidelines recommend a systematic calibration to all users to establish the range, sensitivity, accuracy, precision, and inter-unit variability. There are two different distinguishable levels of calibration: (1) Unit calibration: The internal reliability of the accelerometer sensors across multiple units; and (2) Value calibration: The conversion of accelerometer output into more meaningful information, such as EE or time spent in moderate intensity physical activities which would give more clarity to patients or healthcare professionals [17].

Unit calibration is done to reduce inter-instrument variability and to ensure that individual activity monitors are correctly measuring the acceleration to which they are exposed. Such calibrations can be done for both static and dynamic conditions. The latter can be done with a mechanical shaker across a range of standardized accelerations and frequencies [7,18–21]. Unit calibration is still advised before deployment in actual physical activity measurement to check for any malfunctions, even though contemporary devices with micro-electromechanical accelerometers have initial unit calibration performed at the factory that should remain calibrated for the lifespan of the device [14]. However, as these mechanical shaker studies are not generalizable to free-living conditions, sensor output is often calibrated during standardized activities such as walking on a treadmill or by positioning monitors on the right and left side of the body [21–23].

#### 2.1.1. Effect of Sensor Position

The effect of sensor position on sensor output has been studied with the first generation accelerometers. Positional influences of the accelerometer around the hip were assessed by Jones *et al.* [22]. They positioned accelerometers at three different locations at the right hip, and made subjects walk at 3 mph (4.8 km/h) on a treadmill. They found significant differences in placement for the 1D accelerometer, but no differences for the multidimensional (3D and bidirectional) accelerometers. Welk [17] commented on these results that the multidimensional sensors tested by Jones *et al.* were probably less vulnerable to position differences.

In a more recent study on inter-instrument reliability, Powell *et al.* [24] placed eight 3D activity monitors of the same brand on their subjects: four on the left hip and four on the right hip. During rest and low intensity trials no significant between-unit differences for activity counts were identified. Significant differences were detected, however, during vigorous-intensity trials and relatively high variations were evident during the sit-to-stand task. The findings concerning low intensity activities of Powell *et al.* and Jones *et al.* suggest that there may be an interaction effect between sensor position and activity intensity. This position-intensity effect was also observed by Nichols *et al.* [21] who placed a 3D accelerometer inside a pouch securely fastened to the body with an elastic waist strap on the right and left hip of their participants while they walked on a treadmill. Intra-class correlation coefficients (ICC) of the vector magnitude of the right *vs.* the left sensor declined from 0.87 to 0.73 for respectively walking (3.2 km/h) and running (9.7 km/h). Nichols *et al.* discussed that the lower correlation during running might be caused by the skewed vector magnitude values. These findings on sensor position lead to the hypotheses:

H1: The position of the sensor around the waist affects sensor output.
H2: The effect of sensor positions around the waist on sensor output is mediated by the activity intensity.

#### 2.1.2. Effect of Sensor Mounting

The effect of sensor mounting was observed by Bosch *et al.* [25], who reported that the differences of sensor output from the activity sensor in their study was caused by the two different types of sensor pouches used in several free living conditions. They attributed this finding to the different sensor orientations (wearing the sensor horizontally or vertically). However, the activity sensor used in this study is a 3D accelerometer with equal axes sensitivity. Therefore, it is unlikely that orientation caused the effect in sensor output. Rather, the use of two different types of pouches is more likely to have affected the accelerations measured by the sensors, possibly due to tightness of fit.

A somewhat similar situation can be found in Paul *et al.* [26]. Here, the authors compared two different brands of activity monitors while worn together on an elasticized belt in free living conditions. The authors found a significant lower number of counts per day by the sensor attached to the belt with Velcro compared to the one directly looped through the belt. They concluded that a conversion factor was needed to compare the two brands, due to the proprietary nature of the algorithms on the sensors, while neglecting mounting as factor. While we do not claim we have found the cause for the findings in the aforementioned studies, we do believe that mounting needs to be considered as a factor that affects sensor output. This leads us to our final hypothesis:

H3: Mounting of a sensor with a tighter fit to the body will produce higher sensor output.

## 3. Study Overview

In order to test our hypotheses, we have conducted a two-staged study. First, we performed a calibration study to study the quality of our sensor. Second, we studied the effects of usage on sensor output by focusing on: (1) sensor location and (2) type of mounting, in a laboratory study with healthy subjects.

## 4. Calibration Experiment

### 4.1. Sensor

**Figure 1.**
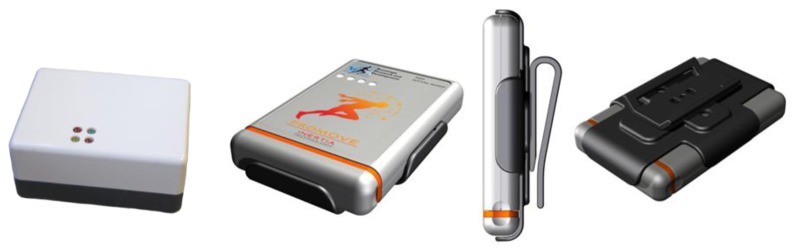
Images of the ProMove2 (**left**) and ProMove3D (**right**).

Recently, a new 3D accelerometer physical activity sensor has been introduced: the ProMove3D (63 × 96 × 16 mm, 67 g, Inertia Technology, Enschede, The Netherlands, Figure 1). This monitor has a 3D MEMS inertial sensor (LIS3LV02DL, ST Microsystems, Geneva, Switzerland) which can provide real-time output of raw 3D accelerometer up to 200 Hz with amplitude range of −6 to +6 g, and can run embedded software protocols for example to output activity counts per minute. The ProMove2 (65 × 50 × 30 mm, 70 g; which is used in this study, is the developer model of the ProMove3D containing the same 3D MEMS inertial sensor, see Figure 1. The ProMove 2 and its successive model the ProMove3D have already been implemented in a number of telemonitoring studies [25,27–29].

Activity monitoring sensors have to be sensitive to accelerations that occur during normal human movement. According to Bouten *et al.* [30], body-fixed accelerometers placed at the waist level should be able to measure an amplitude range of about −6 to +6 g and should measure frequencies up to 20 Hz. These conditions are met by the ProMove2 specifications. The embedded software on the ProMove2 calculates aggregated accelerometer values similar to Bouten *et al.* [30]. These Integral of the Modulus of the Accelerometer output (IMA) values are calculated per minute in metric units (10^−3^ m/s^2^), according to Equation (1), with sample frequency *f_s_* = 100 Hz and time interval T = 60 s. Because of the embedded implementation of the IMA algorithm, the ProMove2 uses a high-pass filter by subtracting a moving average filter based on the last second (100 samples, 100 Hz) from the signal, whereas Bouten *et al.* applied a band-pass filter with cut-off frequencies at 0.11 and 20 Hz:

(1) $$\text{IMA}=\frac{1}{f_{s}T}\sum_{n=n_{0}}^{n_{0}+f_{s}T}\left|a_{x}\left[n\right]\right|+\left|a_{y}\left[n\right]\right|+\left|a_{z}\left[n\right]\right|$$

### 4.2. Method

**Figure 2.**
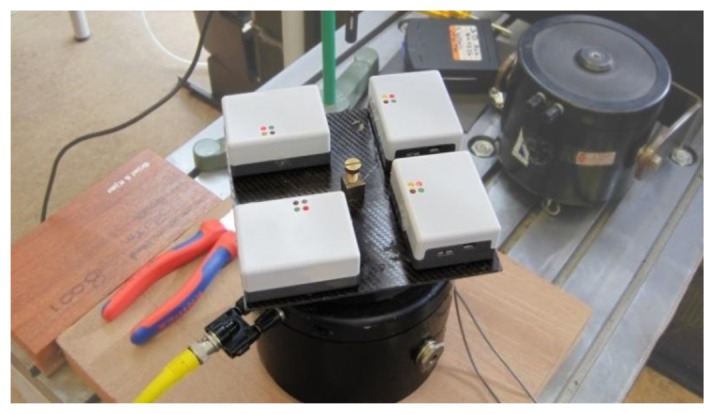
Setup of the sensors on the mechanical oscillator. Four ProMove2 sensors were securely fastened to a mechanical oscillator (Vibration Exciter, type 4809, Brüel & Kjær).

Four ProMove2 sensors were securely fastened to a mechanical oscillator (Vibration Exciter, type 4809, Brüel & Kjær, Nærum, Denmark) using non-damping materials, see Figure 2. The platform was oscillated at three different frequencies (6.67 Hz; 13.45 Hz; 19.88 Hz) within the range of human physical activity for which an activity sensor should be sensitive, according to Bouten *et al.* [30]. Sensors were oscillated for 5 min per frequency, and for each of the three sensor axes, resulting in nine conditions.

The applied acceleration of each condition was measured by the calibrated mechanical oscillator and expressed by an RMS value (RMS = √(1/*n*) ∑*_n_* (*a_x_*^2^ + *a_y_*^2^ + *a_z_*^2^) in 10^−3^ m/s^2^). The ProMove2 measured 3D accelerometer data at 200 Hz. This was converted to RMS values, after removing start/stop effects, by only including the steady state of each condition, and removing gravity. The accuracy of the ProMove2 was evaluated by comparing its RMS values to the RMS of the calibrated mechanical oscillator.

### 4.3. Results

The accelerations measured by the ProMove2 showed that the factory calibration was accurate for all three sensor axes on all three tested sensors. The calculated RMS of the ProMove2 sensors was 4%–7% higher than the RMS of the calibrated oscillating platform and had a low variability between individual axes, indicating high accuracy of the sensors, see Table 1.

### 4.4. Conclusions

The ProMove2 sensor is reliable for measuring accelerations within the frequency range of human movement. No manual calibration is needed, as the factory calibration is sufficiently accurate. Therefore, we could use the sensor in the next stage of the study.

## 5. Laboratory Study

Our hypotheses on the effect of sensor position and method of mounting were tested in a laboratory setting.

### 5.1. Subjects

A convenience sample of ten healthy subjects (five male and five female) participated in the study. The physical characteristics of the subjects are presented in Table 2.

### 5.2. Method

To study the effect of position of the sensor around the waist on sensor output (H1) and a mediating effect of activity intensity (H2), the subjects performed a number of activities for 5.5 min per activity with a 30 s rest period between each in the following order: walking on a calibrated motorized treadmill at four different walking speeds (3, 4, 5, and 6 km/h); slow jogging at the treadmill at 8 km/h; performing a series of predefined deskwork tasks, for example typing, taking a book from a shelf, reading a book, and making a phone call; and walking through a corridor at a comfortable walking speed (CWS). The activities are common daily activities and can be well controlled. Before the experiment, subjects walked for several minutes on the treadmill to get acquainted with treadmill walking.

**Figure 3.**
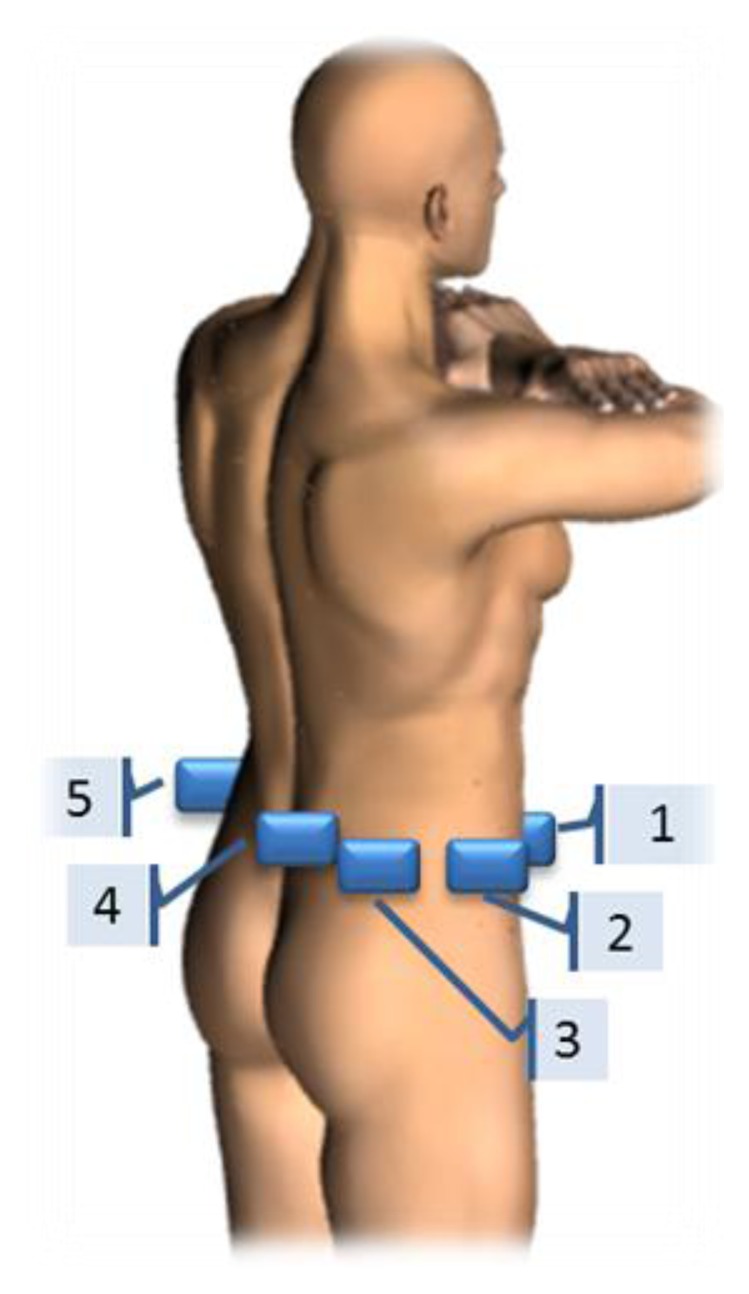
The five sensor locations around the waist. Sensor location 1 = Right hip anterior position; 2 = Right hip most lateral position; 3 = Right hip posterior to position 2; 4 = Sacrum position; and 5 = Left hip most lateral position.

Each subject wore five ProMove2 activity sensors simultaneously, at specific locations around the waist, as shown in Figure 3. These locations correspond to positions often reported in literature. The sensors were worn by the subjects in specially made tight fitting pouches which were securely mounted on an elastic waist belt. This resulted in minimal movement between the sensor and the elastic waist belt. The belt was not removed in between the different types of activities and each device was worn on the same location for all subjects. Subjects were not instructed in their choice of clothing and shoes.

To evaluate the effect of method of mounting on the accelerations measured by the sensors (H3), two of the subjects repeated the test protocol with the same sensors in the same sensor locations, but in commercially available pouches (Exilim leather pouch, Casio, Tokyo, Japan), in which the ProMove2 itself had a tight fit. However, the commercially made pouches were more loosely fitted to the elastic waist belt than was the case for the specially made pouches.

### 5.3. Analyses

Raw accelerometer data were checked for abnormalities. The first and last 20 s of each 5.5 min activity interval were deleted to exclude start and stop effects. The raw acceleration data was then converted to IMA values per minute (Equation (1)). The statistical analyses were done using SPSS (SPSS Statistics, Version 19, IBM, Armonk, NY, USA).

First, position effects on the homogeneity of variances of minute-by-minute IMA values were tested by Levene's test. Equal variances indicate that the reliability of IMA values are not dependent on sensor location. Variances between the different sensor locations are assumed unequal when p < 0.05. Degrees of freedom are given by the F statistic. Second, a two-way repeated measures analysis of variance (ANOVA) with Bonferroni corrected pairwise comparisons was used to determine differences in mean IMA values per activity, due to the type of activity, the sensor location and its interaction-effects. The alpha level was set at p < 0.05 for all tests. Contrasts were performed with all sensor locations compared to sensor location 2 (see Figure 3), as that is the preferred location from a usability perspective in free-living studies. And all activity types were compared to the CWS as this type of physical activity resembles free-living walking conditions the best. Descriptive statistics of the mean IMA value per activity show the effect of the tightness of fit of the sensors at the different sensor locations and at all types of activities.

## 6. Results

### 6.1. Reliability of Raw Accelerations

**Figure 4.**
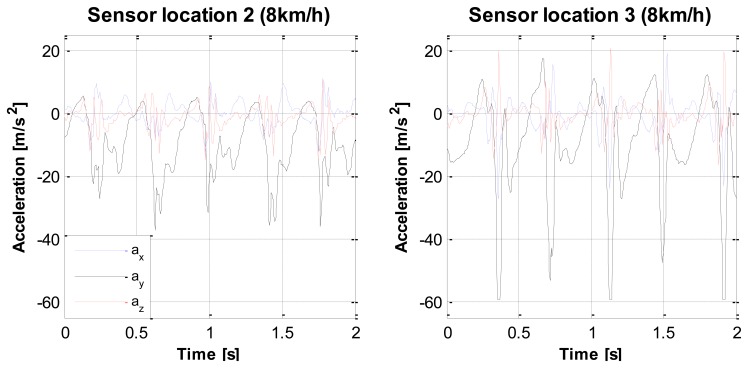
Example of clipping. Example of raw accelerometer data, while jogging 8 km/h. (**Left**), sensor location 2—no clipping. (**Right**), sensor location 3—clipping on one axis at 6 g.

When checking the data for abnormalities, we found that sensor clipping occurred during the jogging activity (8 km/h at the treadmill), as shown in Figure 4. Clipping means that the true accelerations exceeded the sensibility range of the sensor, which was set at 6 g. Clipping was always present at sensor location 3 (right hip posterior position) and often at sensor location 4 (sacrum), at the vertical axis. Due to this clipping, IMA values of jogging are an underestimation of the true accelerations to which the sensors were exposed. Although this phenomenon was present, no correction for the clipping was done in the analysis that followed, as this would also not be done in uncontrolled free living settings.

Minute-to-minute variability of IMA values was analyzed by comparing the percentage of variation within subjects during each steady state with the mean IMA per condition, which varied from 0.8% to 61%, see Table 3. Relative high variances are evident in the deskwork task as this consisted of multiple small tasks making it more prone to minute-to-minute variations. Low minute-to-minute variability can be seen in the treadmill walking and during LGW. Levene's test for homogeneity showed that minute-to-minute variances did not significantly differ between sensor positions of individual activities.

### 6.2. Usage Effects on Sensor Output

**Figure 5.**
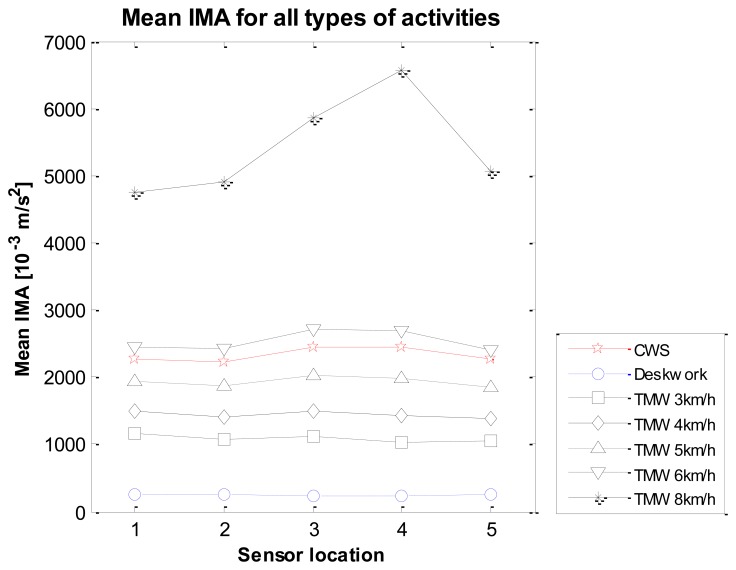
Mean IMA values for sensor locations 1–5, showing different lines for each type of activity (n = 10). TMW = treadmill walking; CWS = comfortable walking speed.

Both main effects (sensor location and type of activity) and their interaction effects were tested with a two-way repeated measures analysis of variance test. Mauchly's test indicated that the assumption of sphericity had only been violated for the main effects of type of activity, χ^2^ (20) = 98.6. Therefore the degrees of freedom were corrected using the Greenhouse-Geisser estimates of sphericity (ε = 0.32) for the main effect of type of activity. Figure 5 shows the mean IMA values from the group with the sensor locations on the X-axis and a line per activity type.

#### 6.2.1. Effect of Type of Activity

**Figure 6.**
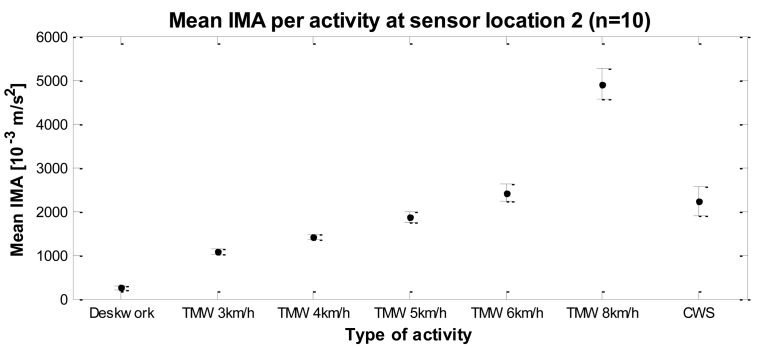
Error bars with 95% confidence intervals for mean IMA values per activity, at sensor location 2 (n = 10); TMW = treadmill walking; CWS = comfortable walking speed.

There was a significant main effect of the type of activity (*F*(1.93, 17.3) = 252.3, p < 0.001): higher walking intensities result in larger accelerations resulting in higher IMA values. This can also be seen in Table 4 and Figure 6 that show the mean IMA values with confidence intervals for each type of activity measured at sensor location 2. Contrasts revealed that IMA values of all activities except treadmill walking at 6 km/h were significantly different from walking at CWS. IMA values of deskwork, 3 km/h, 4 km/h and 5 km/h were lower than CWS, respectively *F*(1, 9) = 152.4, r = 0.97, p < 0.001; *F*(1, 9) = 64.2, r = 0.94, p < 0.001; *F*(1, 9) = 34.4, r = 0.89, p < 0.001; and *F*(1, 9) = 7.9, r = 0.68, p = 0.02. IMA values of treadmill walking at 8 km/h were higher than CWS, *F*(1, 9) = 141.8, r = 0.97, p < 0.001.

#### 6.2.2. Effect of Sensor Location

**Figure 7.**
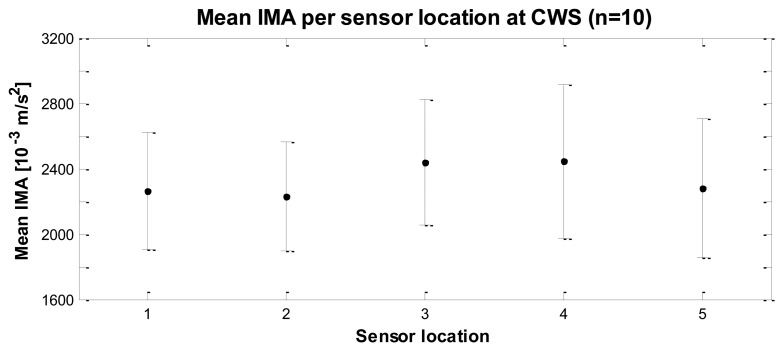
Error bars with 95% confidence Intervals for IMA values from all 5 sensor locations, at comfortable walking speed (CWS).

All subjects wore five sensors simultaneously on an elastic waist belt, which makes it possible to study the effect of the sensor positions on the IMA values. Figure 7 gives the mean IMA value and the confidence intervals for each sensor location during CWS. There was a significant main effect of the sensor location on the mean IMA value, *F*(4, 36) = 49.2, p < 0.001. Contrasts revealed that only the IMA values of sensor locations 3 and 4 were significantly higher than sensor location 2, respectively *F*(1, 9) = 165.7, r = 0.97, p < 0.001; and *F*(1, 9) = 87.5, r = 0.95, p < 0.001. Mean IMA values for sensor locations 2, 3 and 4 and the minimum and maximum values were: 2227 [1426–3089], 2439 [1405–3277], and 2442 [1381–3730]. These results support H1: The position of the sensor around the waist affects sensor output.

#### 6.2.3. Interaction Effect of Type of Activity and Sensor Location

**Figure 8.**
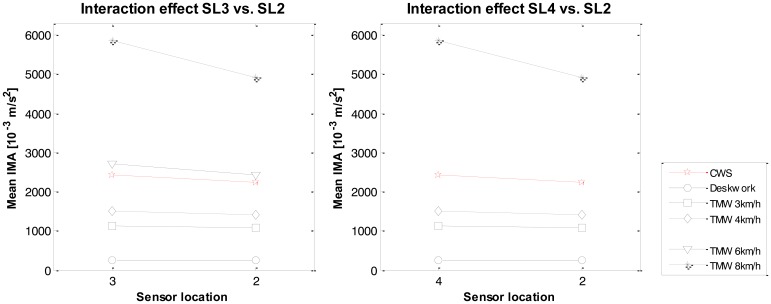
Interaction effects of sensor location and type of setting. Mean IMA values of all 10 subjects for the significant interactions. (**Left**), Location 3 *vs.* 2; (**Right**), Location 4 *vs.* 2.

The interaction effect between the type of sensor location and the type of activity was significant, *F*(24, 216) = 35.9. This indicates that the sensor location had different effects on the IMA values, depending on the type of activity. To break down this interaction, contrasts were performed comparing all sensor locations to their baseline (location 2) and all types of activities to their baseline (CWS). An overview of the significant interaction effects is given in Table 5, which shows that sensor location 1 *vs.* 2 had only one interaction-effect for treadmill walking at 4 km/h *vs.* CWS, in which the difference between location 1 and location 2 is smaller at CWS than at TMW 4 km/h. Sensor locations 3 and 4 showed interaction effects for almost every activity, indicating that the effect of sensor location is strongly affected by the type of activity one performs, both with low intensity activities (deskwork) and with high walking intensities. Finally, no interaction effects were found for sensor location 5 *vs.* 2, which is in line with the non-significant contrast for the main effect of sensor location 5 *vs.* 2. In both graphs of Figure 8 it can be seen that the IMA values at sensor location 3 and 4 increase more at higher intensities than the self-selected walking speed, compared to sensor location 2. These results support H2: The effect of sensor positions around the waist on sensor output is mediated by the type of activity.

#### 6.2.4. Effect of Mounting

The study for the effect of mounting was explorative with only two subjects (A and B). For both subjects the pouches which were loosely fitted to the waist belt had lower IMA values than those of the more securely fitted pouches. The only exception is the deskwork of subject A. With increased walking speeds on the treadmill the effect of the different mounting methods was found to be stronger at all sensor locations. However at sensor location 2 the effect was minimal.

**Figure 9.**
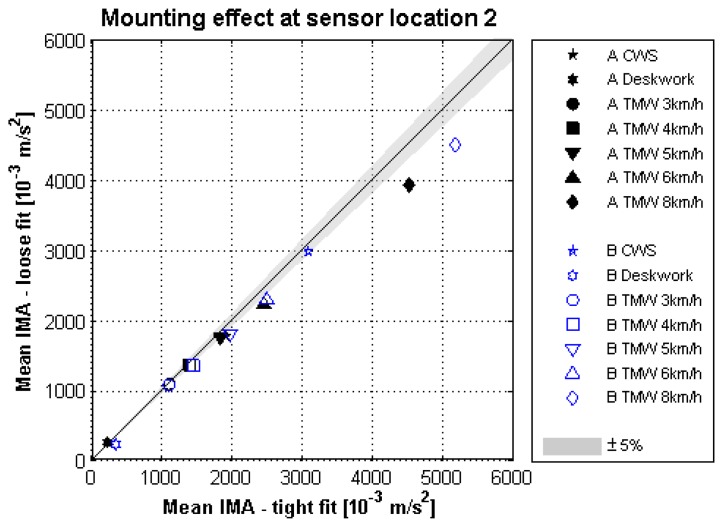
Effect of mounting on IMA values for various activities measured at sensor location 2, for two subjects (A and B, n = 2). IMA values measured with the Exilim pouch (loose fit) *vs.* the more securely fitted pouch.

At this position the IMA values due to the loosely fitted pouch were 2% lower at 3 km/h and 15% lower at 8 km/h compared to the more securely fitted pouch. Finally, at the comfortable walking speed the effect due to mounting at sensor location 2 was 7% and 3% respectively, see Figure 9. These results provide tentative support for H3: Mounting of a sensor with a tighter fit to the body will produce higher sensor output.

### 6.3. Estimated Effect of Position on Free Living Data

By taking a few assumptions, the accumulated effect of position on data of a normal day of free living can be estimated. If we assume that an average monitoring day consists of 14 h (=840 min) of wear time [31], during which adults accumulate 7,473 steps per day [32], if we assume an average of 113 steps per minute [33] this takes about 66 min at an intensity level corresponding to the CWS condition in this study. Finally, we assume that the remainder of the day (non-stepping time) is not affected by sensor position. In Table 6 below we can see the effect of sensor positions 3 and 4 with respect to position 2 on the mean daily activity. When applying the assumptions to the mean daily activity in IMA of a healthy control group in the study of Tabak *et al.* [34]. If we replace 66 min (8%) by the average IMA value for CWS at sensor position 2 and calculate the average IMA for the remaining non-stepping time in order to keep the mean daily activity the same with the reference group, we can see that sensor positions 3 and 4 increase the mean IMA value per minute from 1,162 to 1,179, resulting in an increase of the mean daily activity score with 1.4% and 1.5% respectively.

## 7. Discussion

This study has shown that sensor position affects sensor output. Moreover, we have shown that there is an interaction effect between sensor position and type of activity. This makes it impossible to compare free-living studies that applied different sensor positions, due to the inherent nature of free-living behavior, from which it is unknown which types of activities were performed. Finally, we have estimated the position effect on free-living activity monitoring.

In order to increase the reliability, and to reduce the variability of sensor output, instructions for fastening activity sensors should consequently promote the same position on the body. The results from our study indicate that the most lateral position on a waist belt favors all other positions. First, it is a user-friendly position. If the sensor hampers free movement (a person may touch it with a moving arm), the best alternative is to position the sensor slightly more forward on the belt (to a more central position). This has a minimal effect on sensor output, but still creates a large range where the sensor can be worn with high data-reliability. Second, the lateral position on a waist belt showed no clipping during jogging at 8 km/h, while the sensor positions on the back (sensor locations 3 and 4) did. Finally, our exploration of how tightly a sensor should be fitted to the body suggested that for the best results they should be fitted as tightly to the body as possible. Such a tight fit can be facilitated by providing mounting material that ensures this tight fit, such as an elastic waist belt or clips that create a firm connection to a waistband. This will not hamper the participants' freedom to move as they normally would.

The results of this study enable researchers that are interested in studying activity behavior by means of activity sensors to create a more reliable data set. If they make their participants adhere to the instructions on how to wear an activity sensor, they will be provided with a data set that more closely resembles reality than with a data set that is generated by activity sensors placed at other parts of a waist belt. This means that researchers should also instruct all of their participants to wear their activity sensor on the most lateral position of a waist belt, or should position the sensors there themselves. Finally, when reporting studies that are based on activity monitor data, authors should report both the position where the activity monitor is placed, as well as the method of mounting (tightness of fit e.g., by a belt clip, Velcro, or attached to an elastic belt).

Besides the scientific gains of this study, our conclusions are also relevant for the developers of (electronic) health interventions that utilize activity sensor data. As we already mentioned in our introduction, such interventions are increasingly geared towards personalized feedback and based upon personal activity data. As a result, flaws in these data have a direct impact on the quality of the intervention, and indirectly on a patient's health. Therefore, positioning an activity sensor at the most optimal place on a patient's clothing is of great importance and these interventions should come with clear and explicit instructions that specify how to place the activity monitor to the side of the hip.

One could state that the evolution of activity monitors into more lightweight devices would also result in a reduction of position and mounting effects on accelerometer data due to the reduction of inertia. Such a shift has also been expected when transitioning from 1D to 3D sensors. However, our study has shown that in the latter case, this transition has not solved the problem. We are therefore cautious in accepting the thesis that a similar effect will occur when evolving into lightweight activity monitors, and urge the community to first study the effects of position and mounting on lightweight activity monitors carefully.

### Limitations

The subjects that participated in this study were a convenience sample. They were young, healthy and had no problems walking on a treadmill at the different walking speeds. As a result, the findings of this study can only be generalized with 100% certainty towards the group of healthy young adults. In order to assess the effect of position and mounting on sensor data for other groups (e.g., children, the elderly, and people with walking difficulties), this study should be repeated with a subject sample, representative for this group. That being said, we think that the results of this study show that position and mounting do affect sensor data and we think that this will also be the case for other populations. However, the size of the effects may differ somewhat per population.

We had a subject sample of only two persons for studying the effect of mounting on sensor data. This has limited our possibilities for statistical analysis and hampers generalization. We think the results for mounting should be interpreted as explorative and highlighting the need for further research. We think we have shown that mounting may have an effect during studies which utilize activity monitors, and should be factored in when determining the quality of data.

We have not included a free living condition in our study as it excludes steady state conditions, limiting comparability of the type of activity among subjects and data variability. We have given a very rough estimate of the combined accumulated effect of position and mounting on free living data. The assumptions for this estimate only include an estimate of time spent in the measured activities and disregards the rich diversity of movements during free living, e.g., during household activities. Future research should delve into this issue.

The estimated effect of position on free living data resulted in only 1.4%–1.5% increase in IMA values on a daily basis if the sensor is worn at a more dorsal position than the most lateral position of a waist belt. This effect can be neglected by researchers, but this increase is based on just the position effect during 66 min of steady-state walking, and it is very likely that the remaining 13 h of monitoring time also consists of sensor position effects, which can increase the main daily score even further. More research on the effect of sensor position is therefore needed. On the other hand, researchers that are only studying steady state walking activities have to factor in the effect of −4% to +12% depending on walking speed and sensor position.

The position and mounting effects in this study are not brand specific. The specifications of the hardware and signal processing will determine how this study results will appear with other devices. Different sensitivity ranges and sample frequencies will probably affect sensor output and thereby also mediate the position and mounting factors. However, if the hardware specifications are sufficient, and the raw accelerometer data is processed in with a similar method as the IMA calculation, our findings are valid.

## 8. Concluding Remarks

In many validation studies, commercially available activity monitors have been validated for their ability to predict energy expenditure, physical activity intensity level, or type of physical activity [11]. Each sensor brand has its own preferred, validated sensor position and method of mounting. We have shown that small position changes or a more tight fit of the sensor have strong effects on sensor output. It is thus to be expected that in past validation studies these factors were not well controlled and various factors might have interfered, resulting in a somewhat ‘blurred’ state of the art. With the recommendations from this study, more reliable and reproducible datasets can be gathered, by reducing variability, which can be used for better predictors of energy expenditure and types of physical activity. Our recommendations are also of great importance for developing health interventions that draw on activity monitor output. As stated by Esliger *et al.* [35], “the quality of information from accelerometers is only as good as the devices themselves.” We strongly agree, but propose the following addition: the quality of information from accelerometers is only as good as the devices themselves and how they are worn.

## Figures and Tables

**Table 1.** Results from dynamic calibration of four sensors on a mechanical oscillator. Calibrated and measured RMS are the mean RMS and its standard deviation.

**Condition**	**Calibrated RMS**	**Mean Measured RMS (n = 4)**					**Mean Difference**

		**Sensor 1**	**Sensor 2**	**Sensor 3**	**Sensor 4**	**Average**	
6.67 Hz	322	341	337	333	333	336	+4%
13.45 Hz	1237	1309	1313	1313	1281	1304	+5%
19.88 Hz	2377	2474	2646	2532	2551	2551	+7%

**Table 2.** Subject characteristics (n = 10).

	**Mean**	**SD**	**Minimum**	**Maximum**
Age (yr)	31	8.5	24	51
Height (m)	1.81	0.08	1.69	1.92
Body mass (kg)	78	12.5	60	96
Body mass index (kg/m^2^)	23.7	3.5	18.0	29.4

**Table 3.** Minute-to-minute variability in IMA values during activities in absolute percentual deviation from the mean IMA for a specific activity, at a specific sensor location and for each subject. n = number of IMA samples. CWS = Comfortable walking speed. SD = Standard Deviation.

**Type of Activity**	**n**	**Mean IMA Variability [%(SD)]**					**Levene's** **Test**	

		**Location 1**	**Location 2**	**Location 3**	**Location 4**	**Location 5**	**F**	**p**
Deskwork	52	58 (34)	59 (34)	61 (35)	59 (35)	60 (34)	F(4,255) = 0.09	0.986
CWS	43	1.2 (1.1)	1.4 (1.4)	1.4 (1.4)	1.4 (1.4)	1.2 (1.3)	F(4,210) = 0.27	0.897
TMW 3 km/h	38	1.1 (0.9) *	1.3 (1.1)	1.8 (1.4)	2.1 (1.3) *	1.8 (1.1)	F(4,185) = 4.10	0.003
TMW 4 km/h	39	1.1 (0.9)	1.2 (0.8)	1.2 (0.9)	1.3 (1.1)	1.4 (1.0)	F(4,190) = 0.57	0.683
TMW 5 km/h	40	0.9 (0.8)	0.9 (0.8)	1.1 (0.8)	1.0 (0.9)	0.8 (0.7)	F(4,195) = 0.77	0.544
TMW 6 km/h	37	1.0 (0.7)	1.0 (0.7)	1.1 (0.7)	1.0 (0.7)	0.9 (0.7)	F(4,180) = 0.43	0.787
TMW 8 km/h	34	2.3 (2.1)	1.7 (1.6)	1.6 (1.6)	2.3 (2.1)	2.0 (1.5)	F(4,165) = 1.12	0.348

* *Post-hoc* analysis using Bonferroni correction, shows significant difference p = 0.004.

**Table 4.** Descriptive statistics of mean IMA values per activity at sensor location 2 in 10^−3^ m/s^2^. TMW = treadmill walking.

**Activity**	**N**	**Minimum**	**Maximum**	**Mean**	**Std. Deviation**
Deskwork	10	180	338	248	51
Comfortable walking speed	10	1,426	3,089	2,227	469
TMW 3 km/h	10	935	1,244	1,070	89
TMW 4 km/h	10	1,286	1,575	1,413	93
TMW 5 km/h	10	1,638	2,162	1,867	158
TMW 6 km/h	10	2,037	2,956	2,418	275
TMW 8 km/h	10	4,123	6,003	4,903	491

**Table 5.** Overview of all significant interaction effects. Contrasts for sensor locations to their baseline sensor location 2 and types of activities to their baseline CWS. CWS = comfortable walking speed; TMW = treadmill walking; r = effect size.

**Sensor Location**	**Type of Activity**		**p**	F*	**r**
1 *vs.* 2	TMW 4 km/h	*vs.* CWS	0.040	5.7	0.62
3 *vs.* 2	Deskwork	*vs.* CWS	<0.001	36.5	0.90
3 *vs.* 2	TMW 3 km/h	*vs.* CWS	0.004	14.9	0.79
3 *vs.* 2	TMW 4 km/h	*vs.* CWS	0.011	10.0	0.73
3 *vs.* 2	TMW 6 km/h	*vs.* CWS	0.036	6.1	0.64
3 *vs.* 2	TMW 8 km/h	*vs.* CWS	<0.001	72.0	0.94
4 *vs.* 2	Deskwork	*vs.* CWS	0.012	9.8	0.72
4 *vs.* 2	TMW 3 km/h	*vs.* CWS	0.006	12.8	0.77
4 *vs.* 2	TMW 4 km/h	*vs.* CWS	0.007	12.2	0.76
4 *vs.* 2	TMW 8 km/h	*vs.* CWS	<0.001	47.8	0.92

* Degrees of freedom for all interaction effects = (1, 9).

**Table 6.** Effect of position on mean daily activity in IMA [10^−3^ m/s^2^]. Duration is given in minutes per day. Cumm. = cumulative IMA value for the given duration per day.

**Scenario**	**Steps**			**Non-Stepping Time** **			**Mean Daily Activity**	
		
	**Duration**	**IMA**	**Cumm.**	**Duration**	**IMA**	**Cumm.**	**IMA**	**%**
Reference group	-	-		840	1,162	976,080	1162 *	100%
Sensor location 2	66	2,227	146,982	744	1,071	829,098	1162 *	100%
Sensor location 3	66	2,439	160,974	744	1,071	829,098	1178.7	101.4%
Sensor location 4	66	2,442	161,172	744	1,071	829,098	1178.9	101.5%

* Reference IMA values are from 21 healthy controls in the study of Tabak *et al.* [34] that used sensor position 2;

** No sensor position effect assumed.
